# The R21 malaria vaccine: Spotlight on policy goals and pathways to African vaccine manufacturing

**DOI:** 10.1371/journal.pgph.0003412

**Published:** 2024-07-08

**Authors:** Olusoji Adeyi, Prashant Yadav, Raj Panjabi, Wilfred Mbacham

**Affiliations:** 1 Resilient Health Systems, Washington, District of Columbia, United States of America; 2 Department of International Health, Johns Hopkins Bloomberg School of Public Health, Johns Hopkins University, Baltimore, Maryland, United States of America; 3 Technology and Operations Management, and INSEAD Africa Initiative, INSEAD, Fontainebleau, France; 4 Department of Global Health and Social Medicine, Harvard Medical School, Boston, Massachusetts, United States of America; 5 Harvard Medical School, Boston, Massachusetts, United States of America; 6 Fobang Institutes for Innovations in Science and Technology, Yaoundé, Cameroon; 7 The Biotechnology Centre, University of Yaoundé, Yaoundé, Cameroon; Ifakara Health Institute, UNITED REPUBLIC OF TANZANIA

Malaria impacts millions of lives, mostly in continental Africa, and especially so in the WHO African Region, where 233 million cases accounted for about 94% of global cases in 2022 [1]. Progress in curbing malaria has somewhat plateaued since 2015 [2, 3] due to factors that include drug and insecticide resistance, poor availability of rapid diagnostic tests (RDTs) and artemisinin-based combination therapies (ACTs), and socio-behavior factors [3]. The approval of two new vaccines against malaria—RTS,S/AS01 vaccine in 2021 and more recently the R21/Matrix-M vaccine [3, 4]—has reenergized the fight against malaria. These vaccines were developed with support from public sector and philanthropic partners, and with contributions from African scientists and communities. They have the potential for high impact and are remarkable additions to the toolkit of malaria control.

## The problem

Malaria is included as a top priority antigen in Gavi’s African Vaccine Manufacturing Accelerator, which emphasizes a clear need for additional suppliers or increased capacity to improve market health [5]. Regarding the R21 vaccine, there is a lack of clarity on what continental policies and external commitments should optimize for in the wake of the COVID-19 pandemic, which revealed strategic perils of African dependence on external manufacturers. Should they only seek to ensure access to affordable and quality-assured R21 vaccines on the continent? Or should they also seek to achieve sustainable African end-to-end manufacturing of the vaccine?

The inequities in COVID-19 vaccine access experienced by African countries generated resentment by African leaders [6]. There is now widespread recognition that a different approach is needed: by developing end-to-end vaccine manufacturing capabilities, the Global South would become less dependent on international producers and the global allocation system [7, 8]. For Africa, it also means emphasizing Africa-based manufacturing, given the freeze on COVID-19 vaccine export from India at the height of the pandemic. Admittedly, just because a vaccine is manufactured in an African country does not guarantee availability of that vaccine to other African countries in a crisis. However, the alternative to Africa-based manufacturing is not reliable benevolence, but contracts, allocations, and donations subject to vetoes and geopolitics beyond the purview of African leadership. Recognizing the need for a new approach includes highlighting responsibilities to which African governments must rise at the country and regional levels through the African Continental Free Trade Area (AfCFTA), the African Medicines Agency, a pooled African medicines procurement mechanism, and investments in manufacturing by African governments and the private sector, to grow the region’s scientific workforce, strengthen its regulatory systems, and create an overall biosciences ecosystem.

The R21/Matrix-M vaccine was designed and developed at the University of Oxford, with funding from public and philanthropic sources. Its Phase-III trials and clinical production were done in partnership with SII. The vaccine contains a saponin-based adjuvant called Matrix-M developed by Novavax. The manufacturing plans for the R21/Matrix vaccine are highly concentrated—SII will produce up to 200 million doses of this vaccine per year at its production site in India. Recent announcements suggest initial progress. SII has partnered with DEK Vaccines, a consortium of Ghanaian pharmaceutical companies led by the private sector, for registration, repackaging, distribution, and the "fill and finish" of R21 [9, 10]. SII also signed a local vaccine manufacturing agreement for R21 with Biovaccines Nigeria Limited [10]. While these announcements signal the intent to manufacture R21 in Africa, there is no rigorous and publicly available framework for their independent and prospective evaluation.

Admittedly, the fastest way to scale the production of a new vaccine is to manufacture it at a single site. We recognize the economies of scale in vaccine production and understand the practicalities of technology transfer to new production sites. At the same time, merely reducing production costs and accelerating timelines to production does not address the strategic goals of achieving sustainable African end-to-end manufacturing.

## The proposition

We posit that policies and financing for R21 manufacturing should concurrently optimize for (i) short-term efficiencies from economies of scale, which includes purchasing from external manufacturers, and (ii) actions to ensure the development of end-to-end manufacturing in Africa, such that the dependence is short-lived. The latter is a variant of industrial policy, such as the United States and Europe practice in areas they consider central to their security.

Expanding the footprint of African manufacturers in the R21 value chain requires a technology transfer approach that meticulously assesses existing and potential production facilities in Africa with the capacity to manufacture the R21 vaccine in the medium term, including the production of the drug substance. While Gavi’s African Vaccine Manufacturing Accelerator (AVMA) has established higher milestone payments for drug substance (DS) production and prioritizes malaria vaccines among its essential antigens [11], achieving a milestone under this for malaria vaccine DS production remains highly improbable without a supplementary effort. Funders, developers, and license holders of the R21 malaria vaccine need to facilitate technology transfer in a manner that maximizes the use of current infrastructure/existing production sites. When such partnerships are created, they should specify the timelines for each step, and not leave them vague or open-ended.

Development Finance Institutions, including the US DFC, IFC, and EIB, have established funding lines for African vaccine manufacturing [12]. In their next round of funding, they should emphasize that projects encompassing the entire value chain, especially those involving the manufacturing of drug substances for high public health impact vaccines like malaria, will be prioritized for financing. Technology transfers between global life sciences companies and African vaccine manufacturers are more likely to happen when the deal terms are competitive and beneficial for both parties. To achieve this for R21, African vaccine manufacturers and their investment partners should quickly develop capabilities so they can offer terms that are as attractive as those provided by major Indian vaccine manufacturers like SII. Similarly, efforts are underway to enhance regulatory capabilities in African countries with emerging vaccine manufacturers to reach WHO’s Maturity Level 3 for vaccine production and batch release.

For African vaccine manufacturing to be sustainable, it is imperative to take actions that ensure African scientists’ and manufacturers’ engagement in the complete value chain of producing new vaccines. This is especially important for diseases that are predominantly endemic to Africa, such as *Plasmodium falciparum* malaria. We call for the development and adoption of a scorecard to independently and prospectively assess how robustly any proposed initiative is positioned to achieve the strategic goal of sustainable African end-to-end manufacturing. The Scorecard would include dimensions such as: (a) alignment with the African Union’s goal of manufacturing 60 percent of Africa’s vaccine needs within the continent by 2040; (b) covenants with dated milestones for progress toward end-to-end capabilities, including explicit strategies for transferring the production of the drug substance or bulk antigen to Africa; (c) a governance structure that does not allow vetoes by any non-African institution, foundation, country, or other entity; (d) enabling access to intellectual property and tech-transfers for African vaccine manufacturers transparently and objectively; (e) commitment of African countries, under the auspices of AU, to procure quality-assured R21 vaccines from such manufacturers even before they achieve price competitiveness with products manufactured outside Africa; and (f) similar commitment of international health financing institutions to finance the procurement of R21 vaccines from such manufacturers. Transparency and rigor are essential for accountability.
